# Clinical significance of machine learning algorithm in predicting PPM during TAVR in small annuli

**DOI:** 10.1007/s12928-025-01215-5

**Published:** 2026-01-05

**Authors:** Yu Mao, Yang Liu, Mengen Zhai, Ping Jin, Wenjing Li, Fangyao Chen, Yuhui Yang, Gejun Zhang, Jian Liu, Yingqiang Guo, Xiangbin Pan, Yongjian Wu, Jian Yang

**Affiliations:** 1 Department of Cardiovascular Surgery, Xijing Hospital, 127 Changle West Road, Xi’an, 710032 Shaanxi China; 2 Department of Clinical Research, Make Medical Technology Co. Ltd, Xi’an, Shaanxi China; 3 Department of Epidemiology and Biostatistics, School of Public Health, Xi’an Jiaotong University Health Science Center, Xi’an, Shaanxi China; 4 Department of Cardiovascular Surgery, Fuwai Hospital, National Center for Cardiovascular Disease, Chinese Academy of Medical Science and Peking Union Medical College, Beijing, China; 5 Guangdong Provincial People’s Hospital, Guangzhou, Guangdong China; 6 Department of Cardiovascular Surgery, West China Hospital, Sichuan University, Chengdu, Sichuan China; 7 Department of Cardiology, Fuwai Hospital, National Center for Cardiovascular Disease, Chinese Academy of Medical Science and Peking Union Medical College, Beijing, China

**Keywords:** Small annuli, Transcatheter aortic valve replacement, Pressure gradient of aortic valve, Effective orifice area, Prosthesis-patient mismatch, Machine learning

## Abstract

Compared with left heart catheterization (LHC), the pressure gradient of an aortic valve (PG_AV_) measured by echocardiography during transcatheter aortic valve replacement (TAVR) in small annuli is overestimated. The purpose of this study was to improve the accuracy of PG_AV_ measurements by echocardiography in small annuli and to evaluate the influence of PG_AV_ on prognosis. The internal derivation cohort included 273 consecutive patients with aortic stenosis and a small annulus (computed tomographic scan showing an annulus circumference < 72 mm or area < 400 mm^2^) who underwent TAVR. Patients completed transthoracic echocardiography (TTE) and LHC measurements during TAVR, and an extreme gradient boosting (XGBoost) algorithm was trained. The primary outcome was a composite end point of all-cause mortality and readmission for heart failure. The mean PG_AV_ level measured by TTE was overestimated compared to the LHC measurement {52.5 [interquartile range: 47.5–57.0] mmHg vs. 42.5 (interquartile range: 38.0–46.0) mmHg, *P* < 0.001}. After adjustments to the XGBoost, the mean PG_AV_ measured by TTE could be significantly improved [Pearson correlation coefficient = 0.94, *P* < 0.001]. Importantly, patients with a predicted mean PG_AV_ ≥ 68.6 mmHg showed a significantly increased incidence of composite end points at 2 years after the procedures (40.7% vs. 16.0%, *P* < 0.001). The XGBoost model could effectively improve the accuracy of the mean PG_AV_ measured by TTE during TAVR, and the predicted mean increase in the PG_AV_ level may lead to a worse prognosis.

*Clinical Trial Registration: *ClinicalTrials.gov Protocol Registration System (NCT05044338).

**Graphical Abstract:**

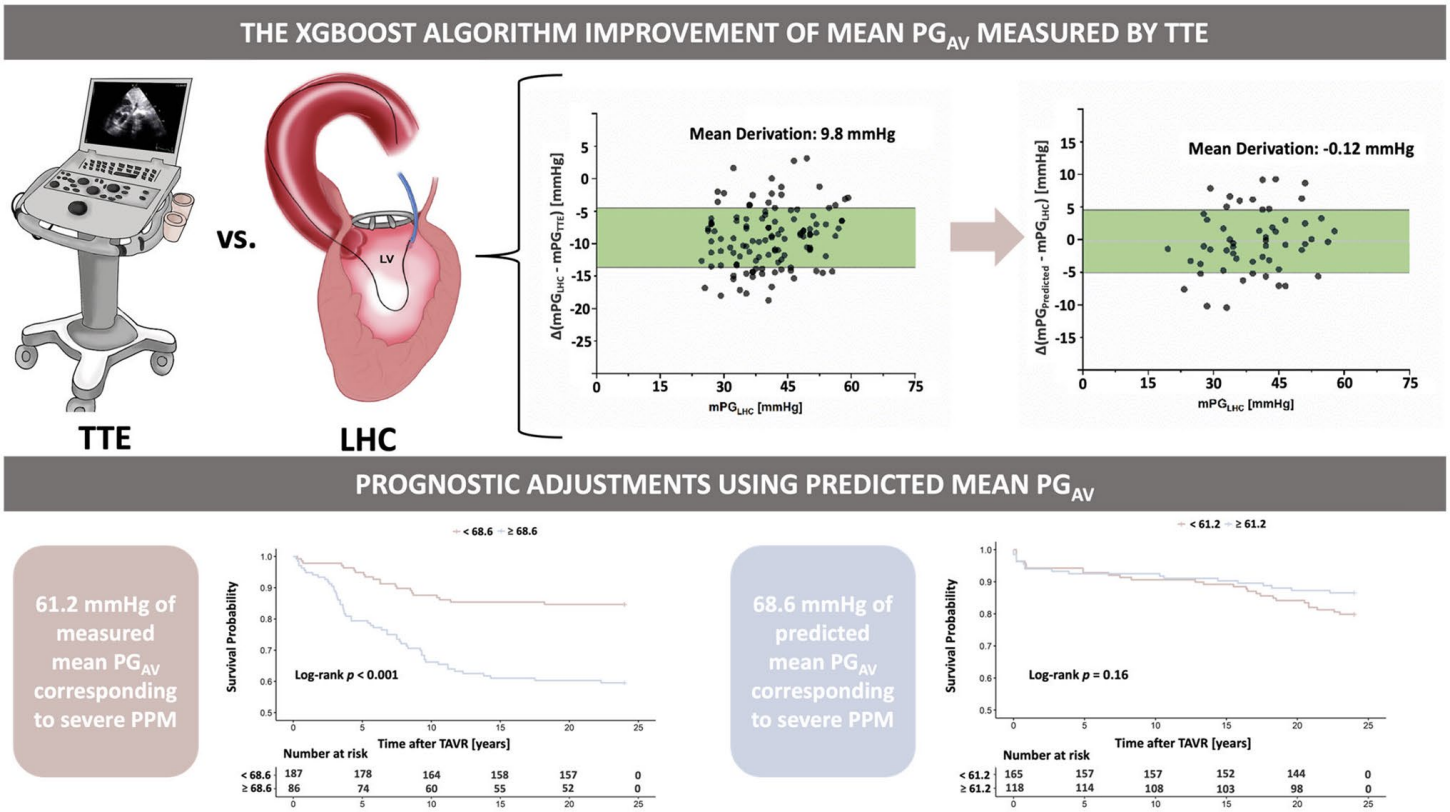

**Supplementary Information:**

The online version contains supplementary material available at 10.1007/s12928-025-01215-5.

The mechanism of prosthesis–patient mismatch (PPM) is that the effective orifice area (EOA) of the implanted valve is smaller than normal, resulting in a significant increase in the pressure gradient and an increased cardiac afterload when cardiac output increases.^1^ Previous studies have shown that the pressure gradient of the aortic valve (PG_AV_) in patients with PPM is higher than that in normal patients and that high PG_AV_ may significantly increase the postoperative mortality [1]. However, compared to mechanical valves, transcatheter heart valves (THV) reduce the risk of PPM [2, 3]. In particular, the performance of the supra-annular self-expandable valves is better than that of the intra-annular self-expandable valves in terms of PG_AV_ [4]. However, for patients with aortic stenosis (AS), the small annulus is considered to be an independent predictor of PPM, so the need to accurately predict the indexed EOA (EOAi) in such patients is urgent [5].

PG_AV_ is usually measured on echocardiography using color flow mapping and aortic valve (AV) morphological assessment. The EOA was obtained by measuring the area of the left ventricular outflow tract (LVOT), the flow velocity time integral, and the flow velocity time integral of AV, respectively. However, the PG_AV_ obtained by echocardiography tends to be overestimated, thus underestimating EOAi [6]. For this reason, when using the modified Bernoulli equation, echocardiography may overestimate the PG_AV_ for several reasons: First, transthoracic echocardiography (TTE) is used to measure the annulus using the long axis section of the LVOT. When there is severe annular calcification in small annuli, the true severity of PG_AV_ cannot be accurately evaluated [7]. In addition, for patients with impaired left ventricular systolic function, the difficulty of measuring PG_AV_ is greatly increased [7]. Left heart catheterization (LHC) is the gold standard for determining aortic and left ventricular pressures and is helpful in evaluating left ventricular systolic and diastolic functions [8, 9]. Therefore, the purpose of this study was to evaluate the effect of postprocedural PG_AV_ on clinical outcomes based on the application of an extreme gradient-boosting (XGBoost) algorithm to improve the accuracy of PG_AV_ after transcatheter aortic valve replacement (TAVR) in patients with small annuli.

## Methods

### Study population

This retrospective observational study included 273 consecutive patients with AS with a small annulus who underwent TAVR from June 2020 to December 2021 at five high-volume centers. Furthermore, an external validation cohort of 118 equally treated patients was provided by 2 centers (enrolled from July 2020 to December 2021). A small annulus was defined as a computed tomography image showing an annular circumference < 72 mm or an area < 400 mm^2^. As recommended by the guidelines, the final decision on whether to proceed with TAVR was made by the local cardiac team [10, 11]. In addition, 23 patients with missing TTE or LHC measurements at discharge and 15 patients with poor computed tomography angiography (CTA) scan quality were excluded. This study complied with the Declaration of Helsinki and was approved by the local ethic commissions. All patients provided written informed consent for procedures and subsequent data collection.

### Definitions and postprocedural measurements

#### PPM

The LVOT diameter was measured from the outer boundary to the outer boundary of the THV directly below the left ventricular boundary of the THV [12]. The pulsed wave Doppler sampling volume was positioned at the THV tip at the same position as the LVOT diameter measurement. For EOAi, PPM is defined as not significant if EOAi >0.85 cm^2^/m^2^; if >0.65 and < 0.85 cm^2^/m^2^, PPM is defined as moderate; and if < 0.65 cm^2^/m^2^, PPM is defined as severe. Additionally, as previously recommended, obese patients (body mass index ≥ 30 kg/m^2^) used a lower EOAi cutoff [13]: if the EOAi was >0.70 cm^2^/m^2^, it was not significant; if >0.55 and < 0.70 cm^2^/m^2^, PPM was defined as moderate; if < 0.55 cm^2^/m^2^, PPM was defined as severe.

#### TTE measurement

PG_AV_ was obtained by subtracting the LVOT pressure from the AV outlet pressure [8, 9].

#### LHC measurement

Under the guidance of X-ray fluoroscopy, the cardiac catheter was inserted into the left ventricular cavity and/or the aorta through the peripheral artery to measure the pressure of the LVOT and the AV outlet.

#### CTA measurement

The main measurements were those for the annular area, diameter, and circumference, the diameter of the sinotubular junction, LVOT, ascending aorta, and the height of the left and right coronary arteries.

### End points

#### Primary performance end point

Procedural success was defined as a successful approach, deployment, and device positioning, and the mean PG_AV_ and EOA were measured by TTE using the continuity equation before discharge, then indexed against the body surface area to define PPM.

#### Primary clinical end point

The primary clinical end point was the composite end point of all-cause mortality after the procedures and readmission for heart failure. Furthermore, the incidence of stroke and related complications (a new-onset permanent pacemaker implant and procedural and device-related complications) was evaluated.

### Statistical analysis

All data were tested for normality and homogeneity of variance. Continuous variables were expressed as mean ± standard deviation or median and interquartile range (IQR). The result of the classified data was expressed as n (%). The Student *t*-test and the Mann-Whitney U test were used to compare continuous normally and non-normally distributed variables, respectively, using the χ^2^ test or the Fisher exact test when appropriate.

In the collinearity analysis, Pearson and Spearman correlation coefficients were calculated. The XGBoost algorithm was chosen as the machine learning (ML) technique of choice for PG_AV_ prediction. Shapley additive explanations values were calculated as the latest measure from cooperative game theory [14] to quantify the contribution of input variables to model predictions [15]. Meanwhile, restricted cubic spline (RCS) analysis of EOAi was performed to obtain the corresponding average PG_AV_ of moderate and severe PPM.

In order to distinguish patients based on prognosis, a cohesive hierarchical clustering algorithm (clustering independent of survival data) was applied. The variables used for clustering were selected by defining the following criteria: (1) Routine measurements of TTE and LHC after TAVR; (2) A well-represented mass of the first 5 dimensions of principal component analysis, defined by the cosine squared (cos^2^). After variable selection, Ward’s minimum variance method was applied to carry out coherent hierarchical clustering.

A simple model of survival after procedures based on the predicted increase in the mean PG_AV_ level was established using the maximum selected logarithmic rank statistic. Survival rates were described using the Kaplan-Meier method, and the hazard ratio was estimated using the Cox proportional risk model.

Bilateral *P* < 0.05 was considered statistically significant. All statistical analyses were performed using SPSS software Version 26.0 (SPSS, Inc., Armonk, NY, USA) and Stata software Version 14.2 (Stata Corp., College Station, TX, USA).

### Results

#### Baseline and preprocedural imaging characteristics

In the internal derivation cohort of 273 consecutive patients with AS with a small annulus, the average age was 73.0 (IQR: 67.0–79.0) years; 52.0% of patients were male; 90.8% of New York Heart Association (NYHA) functional class levels were ≥ III, the average Society of Thoracic surgeons score was 5.10 (IQR: 3.60–7.25) %; and the average n-terminal pro-b-type natriuretic peptide (NT-proBNP) level was 1749.0 (IQR: 1125.0–2368.0) pg/mL (Table 1). Notably, the average EOAi of the internal derivation cohort was 0.46 (IQR: 0.37–0.58) cm^2^/m^2^, and the average annular area was 345.5 (IQR: 325.0–367.5) cm^2^ (Table 2). In addition, there were no significant differences in demographic and preprocedural imaging characteristics between the external validation cohort and the internal derivation cohort (Supplemental Tables 1 and 2).

**Table 1:** Baseline characteristics of the internal derivation cohort

	All Cohorts (*n* = 273)	Cluster I (*n* = 139)	Cluster II (*n* = 134)	*P*-Value
Age, years	73.0 (67.0–79.0)	74.0 (67.0–79.5)	72.0 (66.0–77.5)	0.506
Male	52.0 (142)	51.8 (72)	52.2 (70)	0.799
Body mass index, kg/m^2^	25.0 (23.0–27.0)	25.0 (23.0–27.0)	25.0 (23.0–27.5)	0.766
Body surface area, m^2^	1.68 (1.58–1.81)	1.68 (1.56–1.81)	1.66 (1.56–1.81)	0.623
Diabetes mellitus	24.5 (67)	28.8 (40)	20.1 (27)	0.103
Hypertension	81.0 (221)	84.2 (117)	77.6 (104)	0.320
Dyslipidemia	23.1 (63)	25.9 (36)	20.1 (27)	0.571
Peripheral artery disease	11.7 (32)	14.4 (20)	9.0 (12)	0.215
COPD	6.6 (18)	7.9 (11)	5.2 (7)	0.402
Cerebrovascular disease	5.9 (16)	7.2 (10)	4.5 (6)	0.462
Chronic kidney disease	14.3 (39)	16.6 (23)	11.9 (16)	0.256
Coronary artery disease	31.1 (85)	36.7 (51)	25.4 (34)	**0.034**
Myocardial infarction	4.8 (13)	5.8 (8)	3.7 (5)	0.668
Previous PCI	24.2 (66)	28.1 (39)	20.1 (27)	0.262
Previous CABG	7.3 (20)	8.6 (12)	6.0 (8)	0.380
Atrial fibrillation	16.9 (46)	18.0 (25)	15.7 (21)	0.335
Previous PPI	8.1 (22)	8.6 (12)	7.5 (10)	0.876
NYHA functional class ≥ III	90.8 (248)	97.8 (136)	83.6 (112)	**< 0.001**
STS score, %	5.10 (3.60–7.25)	5.50 (3.90–7.65)	4.55 (3.60–6.40)	0.283
NT-proBNP, pg/mL	1749.0 (1125.0–2368.0)	2414.0 (1570.0 to 3272.0)	1302.0 (802.5 to 1966.0)	**< 0.001**

CABG: coronary artery bypass grafting; COPD: Chronic obstructive pulmonary disease; NT-proBNP: N-terminal pro-B-type natriuretic peptide; NYHA: New York Heart Association; PCI: precancerous coronary intervention; PPI: permanent pacemaker implant; STS: Society of Thoracic Surgeons. Statistical significance was defined as p < 0.05

**Table 2:** Preprocedural imaging assessments of the internal derivation cohort

	All Cohorts (*n* = 273)	Cluster I (*n* = 139)	Cluster II (*n* = 134)	*P*-Value
*TTE and LHC*				
Bicuspid aortic valve	7.0 (19)	6.5 (9)	7.5 (10)	0.790
EOA, cm^2^	0.59 (0.50–0.73)	0.58 (0.48–0.66)	0.62 (0.52–0.74)	0.463
EOAi, cm^2^/m^2^	0.46 (0.37–0.58)	0.40 (0.32–0.54)	0.50 (0.38–0.62)	0.230
≥ Severe aortic stenosis	85.7 (234)	87.1 (121)	84.3 (113)	0.903
Combined with ≥ moderate aortic regurgitation	9.9 (27)	11.5 (16)	8.2 (11)	0.303
Combined with ≥ moderate mitral regurgitation	7.3 (20)	10.8 (15)	3.7 (5)	**< 0.001**
Mean PG_AV_ measured by TTE, mmHg	52.5 (47.0–57.0)	53.5 (49.0–59.0)	51.0 (46.5–56.5)	0.896
Mean PG_AV_ measured by LHC, mmHg	42.5 (38.0–46.0)	41.0 (37.0–44.0)	39.5 (36.0–43.0)	0.875
LVEF, %	50.5 (43.0–56.0)	46.0 (40.5–51.0)	54.5 (50.5–58.0)	**< 0.001**
Peak velocity, m/s	3.7 (2.9–4.4)	3.6 (2.9–4.0)	3.8 (3.1–4.4)	0.620
LVEDV, mL	81.0 (60.0–95.0)	79.0 (57.5–91.5)	83.0 (66.0–103.0)	0.080
LVESV, mL	33.5 (21.0–49.5)	32.0 (21.0–43.5)	36.0 (21.0–55.0)	0.294
Left ventricular mass index, g/m^2^	128.0 (107.5–135.5)	127.5 (111.0–148.0)	130.5 (115.0–151.0)	0.183
Left atrial volume index, mL/m^2^	39.5 (33.5–45.5)	38.8 (33.2–42.5)	40.5 (35.5–47.5)	0.117
Cardiac output, L/min	3.89 (3.34–4.71)	3.55 (3.09–4.29)	4.22 (3.69–4.94)	**< 0.001**
Cardiac index, L/min·m^2^	2.25 (1.82–2.70)	2.09 (1.79–2.42)	2.45 (1.94–2.79)	**0.013**
*Computed tomography angiography*				
Annular area, mm^2^	345.5 (325.0–367.5)	334.0 (323.0–372.5)	352.0 (326.5–370.0)	0.582
Mean annular diameter, mm	21.1 (19.9–22.2)	20.9 (20.2–22.0)	21.0 (20.1–22.3)	0.368
Minimum annular diameter, mm	18.5 (17.4–19.8)	18.4 (17.5–19.4)	18.6 (17.7–19.8)	0.447
Maximum annular diameter, mm	23.7 (22.4–25.0)	23.2 (22.1–24.5)	24.3 (23.1–24.9)	0.440
Annular perimeter, mm	66.3 (64.8–69.6)	65.4 (63.6–68.3)	67.4 (66.2–70.1)	0.201
Annular ellipticity	1.25 (1.15–1.37)	1.27 (1.16–1.35)	1.23 (1.14–1.33)	0.753
Area derived annular diameter, mm	20.9 (19.9–21.5)	20.6 (19.8–21.7)	21.1 (20.1–21.7)	0.449
Perimeter derived annular diameter, mm	21.1 (20.2–22.2)	20.7 (20.3–21.8)	21.2 (20.1–21.9)	0.361
Sinotubular junction diameter, mm	26.1 (24.6–27.1)	25.4 (24.2–27.2)	26.4 (24.7–27.7)	0.110
LVOT diameter, mm	29.4 (27.6–30.4)	29.2 (27.8–30.4)	29.5 (28.5–30.7)	0.447
Ascending aorta diameter, mm	32.3 (29.4–34.9)	32.0 (29.8–34.3)	33.3 (29.9–35.2)	0.373
Left coronary artery height, mm	11.9 (9.8–13.1)	11.8 (10.2–13.3)	11.9 (10.1–13.1)	0.790
Right coronary artery height, mm	14.4 (12.9–15.9)	14.2 (12.6–15.6)	14.5 (13.0–16.0)	0.613

EOA: effective orifice area; EOAi: indexed effective orifice area; LHC: left heart catheterization; LVEDV: left ventricular end diastolic volume; LVEF: left ventricular ejection fraction; LVESV: left ventricular end systolic volume; LVOT: left ventricular outflow tract; PGAV: pressure gradient of aortic valve; TTE: transthoracic echocardiography. Statistical significance was defined as p < 0.05

#### Mean PG_AV_ measurements of TTE and LHC

**Fig. 1:**
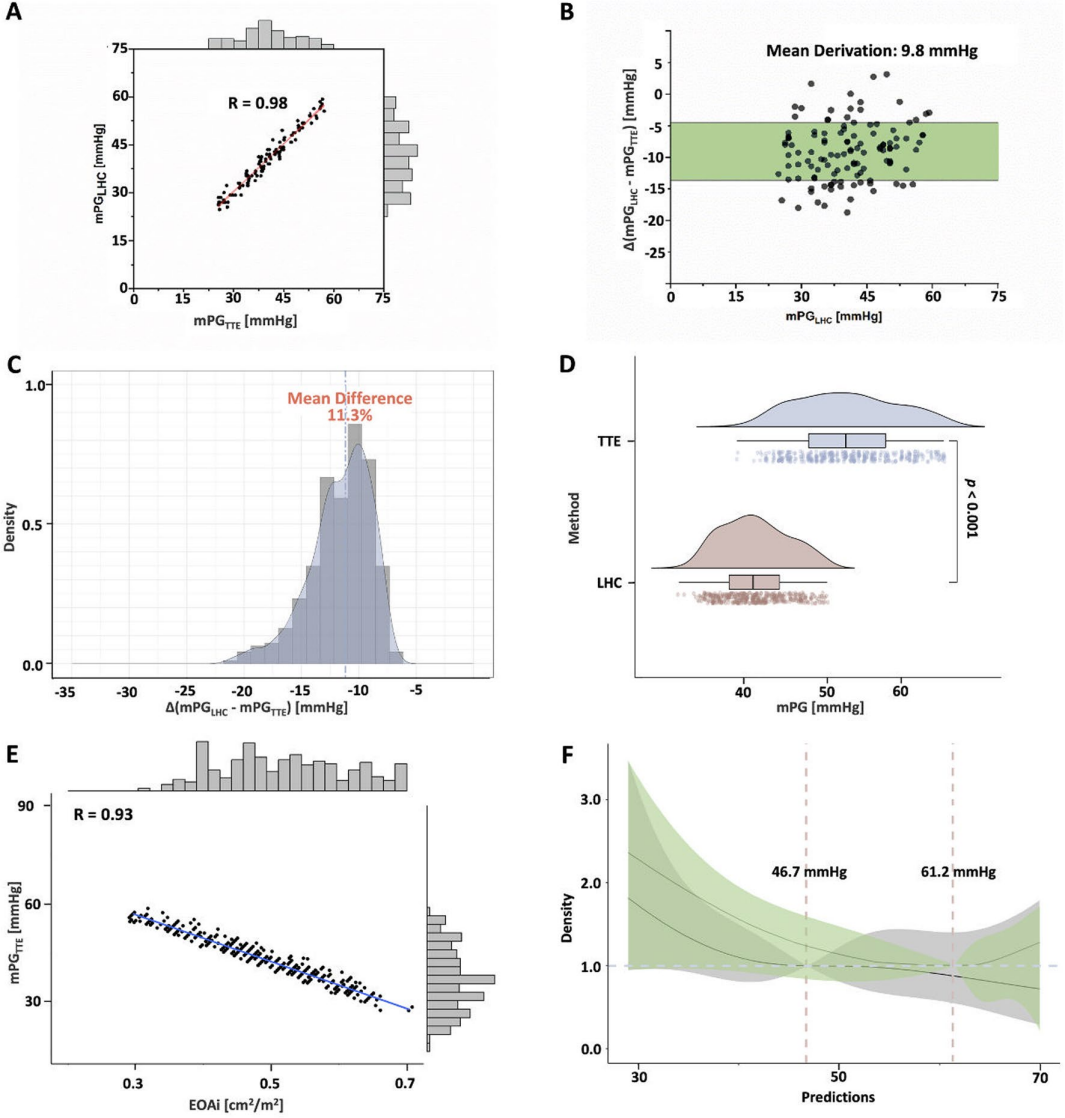
Transthoracic Echocardiography and Left Heart Catheterization Measurement of the Mean Pressure Gradient of The Aortic Valve. (**A**) Strong collinearity between TTE and LHC measurements (*R* = 0.98). (**B**) Mean difference: 9.8 mmHg (95% CI: 4.5, 14.0). (**C**) Median difference range: 11.3%. (**D**) TTE-measured PG_AV_ significantly higher than LHC. (**E**) Inverse correlation between TTE-measured PG_AV_ and EOAi (*R* = 0.93). (**F**) TTE-based cutoff values for moderate and severe PPM: 46.7 and 61.2 mmHg. EOAi: indexed effective orifice area; mPG_LHC_: mean pressure gradient measured by left heart catheterization; mPG_TTE_: mean pressure gradient measured by transthoracic echocardiography

. Linear regression analysis showed significant collinearity in the mean PG_AV_ measured by TTE and LHC, with a Pearson correlation (R) coefficient of 0.98 (*P* < 0.001) (Fig. 1A). Importantly, the mean difference in PG_AV_ assessed by TTE and LHC was 9.8 [95% confidence interval (CI): 4.5, 14.0] mmHg (Fig. 1B), with a median difference range of 11.3% (Fig. 1C). In addition, the mean PG_AV_ measured by TTE was significantly higher than that measured by LHC [52.5 (IQR: 47.5–57.0) mmHg vs. 42.5 IQR: (38.0–46.0) mmHg, *P* < 0.001] (Fig. 1D).

Notably, there was a significant inverse correlation between the mean PG_AV_ and EOAi measures by postprocedural TTE (*R* = 0.93, *P* < 0.001) (Fig. 1E). Therefore, according to the definition of the PPM critical value [13], RCS analysis was used to obtain corresponding mean PG_AV_ cutoff values for moderate and severe PPM, respectively (mean PG_AV_ for moderate PPM = 46.7 mmHg; mean PG_AV_ for severe PPM = 61.2 mmHg) (Fig. 1F).

#### Improvement of mean PG_AV_ by the XGBoost algorithm

**Fig. 2:**
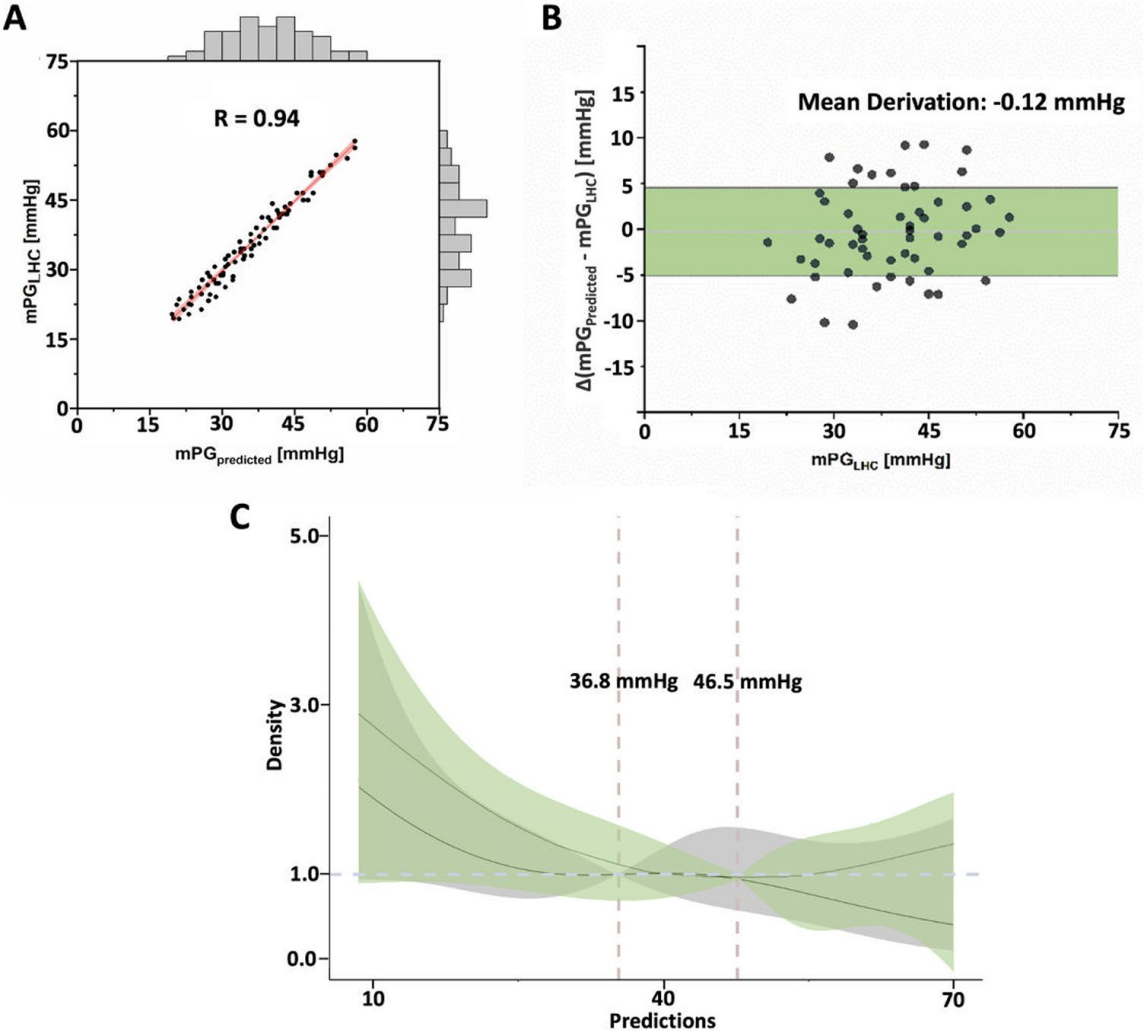
Improvement of the Mean Pressure Gradient of the Aortic Valve by The Extreme Gradient Boosting (XGBoost) Algorithm. (**A**) Strong correlation between XGBoost-predicted and LHC-measured PG_AV_ (*R* = 0.94). (**B**) Mean difference: −0.12 mmHg (95% CI: −5.73, 4.97). (**C**) XGBoost-based cutoff values for moderate and severe PPM: 36.8 and 46.5 mmHg

After obtaining results showing significant differences between the mean PG_AV_ measured by TTE and LHC, the XGBoost algorithm was used to improve the mean PG_AV_ obtained by TTE measurements. Input variables included the following: left ventricular ejection fraction (LVEF), left ventricular end systolic volume, peak velocity, left ventricular mass index, left atrial volume index, mean PG_AV_, EOAi, and STJ diameter. Notably, the mean PG_AV_ predicted by measurement of the XGBoost algorithm had a significant positive correlation with the mean PG_AV_ obtained by LHC (*R* = 0.94, *P* < 0.001) (Fig. 2A). The mean difference between the predicted and the LHC measured for the mean PG_AV_ was − 0.12 [95%CI: −5.73, 4.97] mmHg (Fig. 2B). Importantly, the mean PG_AV_ cutoff obtained using RCS analysis for the moderate PPM critical value was 36.8 mmHg, whereas the mean PG_AV_ for the severe PPM critical value was 46.5 mmHg (Fig. 2C).

#### Clinical outcomes and prognosis evaluation after cohesive hierarchical cluster analysis

**Fig. 3:**
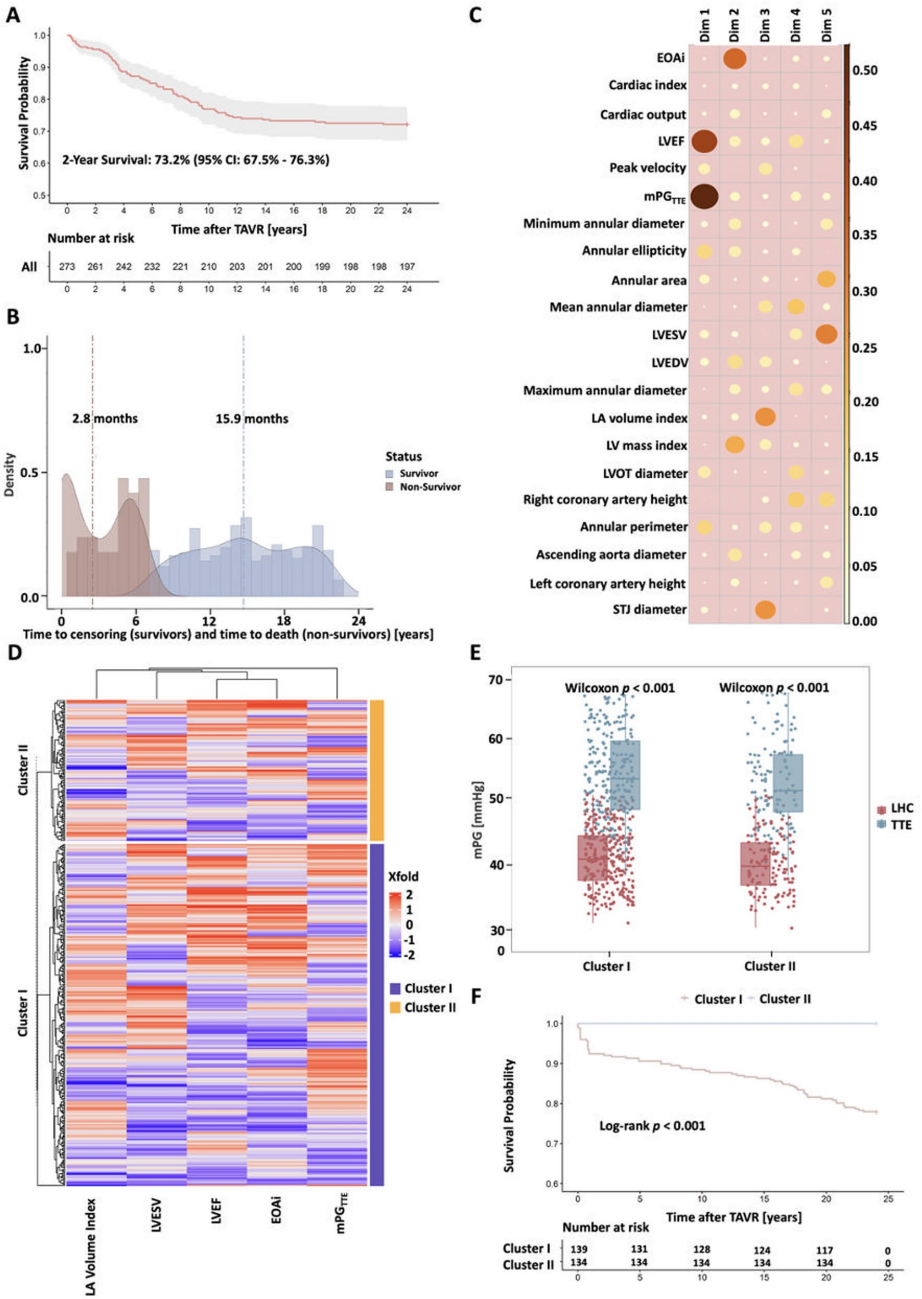
Cohesive Hierarchical Clustering Analysis and Clinical Outcomes Evaluation. (**A**) 2-year composite endpoint incidence: 26.8% (95% CI: 23.7–32.5%). (**B**) Time to 50% mortality: 8.3 months; censoring: 14.9 months. (**C**) PCA representation of candidate variables. (**D**) Heatmap and dendrogram of clustering results. (**E**) Comparison of PG_AV_ between and within clusters. (**F**) Kaplan-Meier survival by cluster assignment. CI: confidence interval; Dim: dimension; LHC: left heart catheterization; LV: left ventricle; LVEDV: left ventricular end diastolic volume; LVEF: left ventricular ejection fraction; LVESV: left ventricular end systolic volume; LVOT: left ventricular outflow tract; TAVR: transcatheter aortic valve replacement; TTE: transthoracic echocardiography

In the internal derivation cohort, 93.4% of patients achieved procedural success (Table 3), the estimated 2-year composite end point incidence range was 26.8% (95% CI: 23.7%–32.5%) (Fig. 3A), and 50% of deaths occurred within 8.3 months after TAVR (Fig. 3B). Importantly, the survival differences among patients were evaluated using the cohesive hierarchical clustering algorithm (Fig. 3C), and the number of 21 candidate variables was reduced to 5 final variables (mean PG_AV_, EOAi, LVEF, left ventricular end systolic volume, and left atrial volume index) for clustering (Fig. 3D). Cohesive hierarchical clustering analysis (Fig. 3C-D) divided patients into two clusters, with key differences and clinical outcomes as follows: Compared with Cluster II, Cluster I showed poorer cardiac function and worse prognosis. specifically, Cluster I had a higher proportion of severe cardiac function impairment (NYHA ≥ III class) and higher NT-proBNP levels, accompanied by more significant left ventricular systolic dysfunction and lower cardiac output/index (detailed data see Fig. 3E). Notably, although there was no significant difference in mean PG_AV_ (measured by TTE or LHC) between the two clusters, the mean PG_AV_ measured by LHC was consistently higher than that by TTE in both clusters (all *P* < 0.001, Fig. 3E). Clinically, Cluster I had a significantly higher incidence of 2-year composite end points than Cluster II (21.6% vs. 0%, *P* < 0.001, Fig. 3F).

**Table 3:** Procedural details and in-hospital clinical outcomes of the internal derivation cohort

	All Cohorts (*n* = 273)	Cluster I (*n* = 139)	Cluster II (*n* = 134)	*P*-Value
*Procedural details*				
Procedural success	93.4 (255)	92.1 (128)	94.8 (127)	0.816
THV type				
Self-expandable valve	82.8 (226)	83.5 (116)	82.1 (110)	0.907
Balloon-expandable valve	17.2 (47)	16.6 (23)	17.9 (24)	0.795
Predilation	78.4 (214)	79.9 (110)	77.6 (104)	0.834
Postdilation	42.5 (116)	36.7 (51)	48.5 (65)	0.230
Conversion to surgery	1.1 (3)	0.7 (1)	1.5 (2)	1.000
Malpositioning	1.1 (3)	0.7 (1)	0.7 (1)	1.000
Annular rupture	0.7 (1)	0 (0)	0.7 (1)	1.000
Device displacement	5.5 (15)	6.5 (9)	4.5 (6)	1.000
Valve-in-valve implant	5.5 (15)	6.5 (9)	4.5 (6)	1.000
Oversizing derived by perimeter ≥ 15%	61.2 (167)	64.0 (89)	58.2 (78)	0.214
Immediate postprocedural mean PG_AV_ measured by LHC	−2.0 (−10.0–6.5)	8.0 (2.0–13.0)	−16.0 (−24.0–9.0)	**< 0.001**
*In-hospital clinical outcomes*				
All-cause mortality	1.5 (4)	2.9 (4)	0 (0)	0.575
Major adverse cardiovascular events	1.5 (4)	2.9 (4)	0 (0)	0.575
Stroke	1.1 (3)	2.2 (3)	0 (0)	1.000
Life-threatening bleeding	2.6 (7)	4.3 (6)	0.7 (1)	0.110
Acute kidney failure	4.0 (11)	5.0 (7)	3.0 (4)	1.000
Major vascular complications	2.9 (8)	2.2 (3)	3.7 (5)	1.000
New-onset PPI	8.7 (24)	15.8 (22)	1.5 (2)	**< 0.001**
Postprocedural mean PG_AV_ measured by TTE	11.0 (6.0–18.5)	17.0 (7.5–28.0)	6.0 (2.0–9.0)	**< 0.001**
Postprocedural EOAi measured by TTE	0.90 (0.62–1.09)	0.72 (0.43–0.99)	1.06 (0.88–1.21)	**< 0.001**

EOAi: indexed effective orifice area; LHC: left heart catheterization; PG_AV_: pressure gradient of aortic valve; PPI, permanent pacemaker implant; THV: transcatheter heart valve; TTE: transthoracic echocardiography. Statistical significance was defined as p < 0.05

**Fig. 4:**
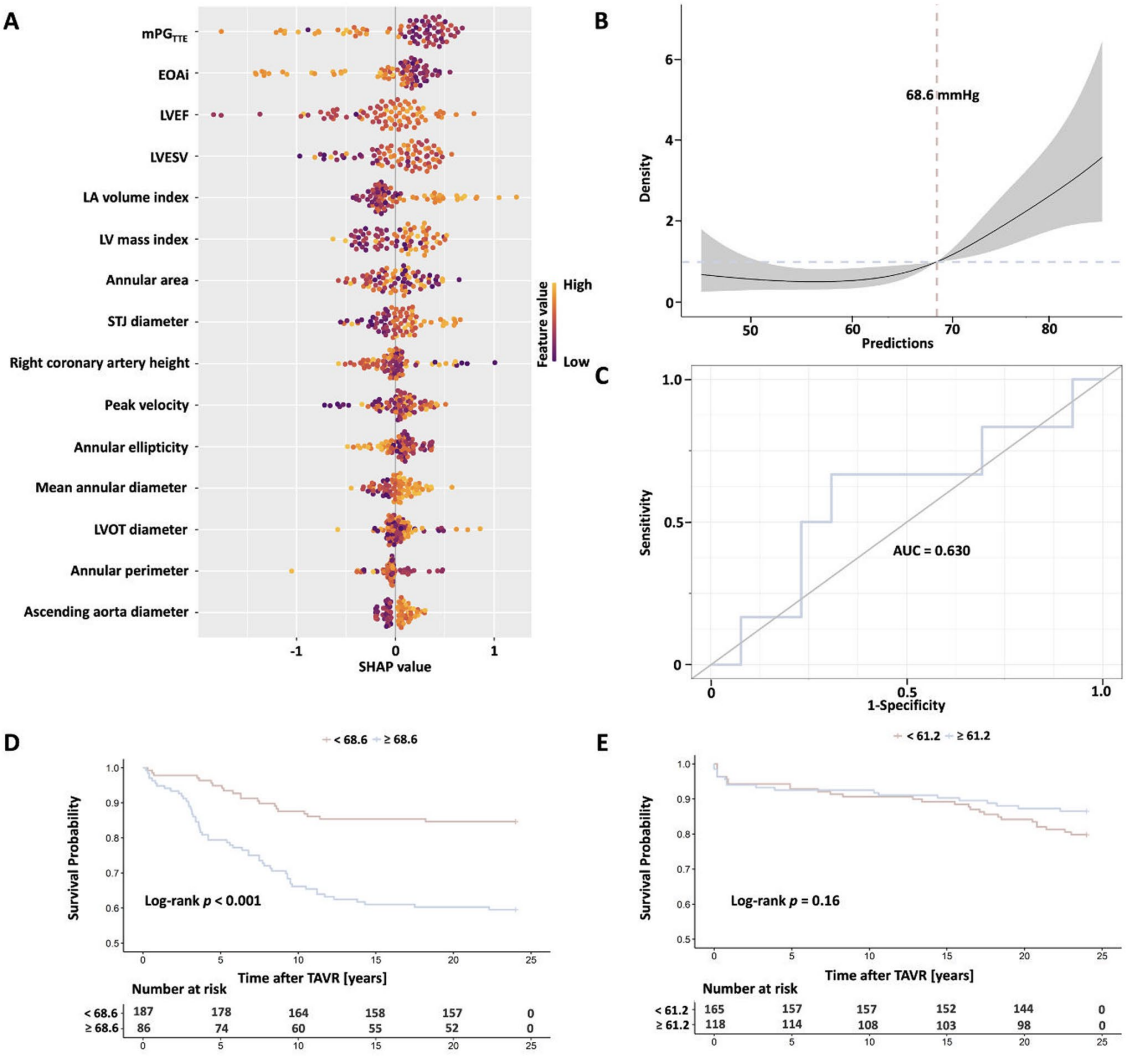
The Survival Model After Transcatheter Aortic Valve Replacement Was Established Based on the Predicted Increase of the Mean PG_AV_ Level Using the Maximum Selected Logarithmic Rank Statistic. (**A**) SHAP analysis of feature importance. (**B**) RCS-derived cutoff for predicted PG_AV_: 68.6 mmHg. (**C**) AUC of the prediction model: 0.630. (D) Kaplan-Meier survival by predicted PG_AV_ (XGBoost) (E) Kaplan-Meier survival by TTE-measured PG_AV_. AUC: area under the curve; SHAP: Shapley additive explanations; STJ: sinotubular junction. *Central illustration*: Predicting mean PG_AV_ based on TTE measurements adjusts evaluation of PPM in patients with AS who have a small annulus and updates prognostic resolution after TAVR

Of interest, to quantify the contribution of input variables to model predictions, Shapley additive explanations analysis was used to evaluate the highest global characteristic importance of mean PG_AV_ predictions. Among them, the global characteristics of mean PG_AV_, EOAi, LVEF, left ventricular mass index, and left atrial volume index were the most significant (Fig. 4A). The survival model was established based on the predicted increase of the mean PG_AV_ level using the maximum selected logarithmic rank statistic. According to the results of RCS analysis, the predicted mean PG_AV_ of 68.6 mmHg was the cutoff value (Fig. 4B). The area under the curve of this prediction model was 0.630, confirming its availability and reliability (Fig. 4C). Compared with patients with a predicted mean PG_AV_ < 68.6 mmHg, patients with a predicted mean PG_AV_ ≥ 68.6 mmHg had a significantly higher incidence of 2-year composite end points (40.7% vs. 16.6%, *P* < 0.001) (Fig. 4D). Notably, a dichotomy of the mean PG_AV_ cutoff value derived from the severe PPM cutoff for the TTE measured above could not significantly distinguish the 2-year composite end point incidence (Fig. 4E).

#### External validation performance

The difference of survival in the external validation cohort was evaluated using the cohesive hierarchical clustering algorithm. Compared with cluster IV, cluster III had a higher proportion of patients who were NYHA functional class ≥ III (100.0% vs. 80.3%, *P* = 0.020) and a significantly higher proportion of those with NT-proBNP [2582.0 (IQR: 1444.0–3371.0) pg/mL vs. 1591.5 (IQR: 1001.0–1771.5) pg/mL, *P* < 0.001)] (Supplemental Table 1). Of interest, cluster III had a significantly higher proportion of patients with ≥ moderate mitral regurgitation (19.1% vs. 7.9%, *P* < 0.001) (Supplemental Table 2). Furthermore, 94.9% of patients in the external validation cohort achieved procedural success (Supplemental Table 3). Notably, there was a significant positive correlation between the mean PG_AV_ predicted by XGBoost analysis and the mean PG_AV_ obtained by LHC measurements (*R* = 0.87, *P* < 0.001) (Supplemental Fig. 1 A). The mean difference between predicted and LHC estimates for the mean PG_AV_ was − 0.69 [95%CI: −2.63, 2.38] mmHg (Supplemental Fig. 1B). The mean PG_AV_ of predictions for moderate and severe PPM critical values obtained using RCS analysis did not differ significantly from the internal derivation cohort (predicted mean PG_AV_ for moderate PPM: 38.2 mmHg vs. 36.8 mmHg; predicted mean PG_AV_ for severe PPM: 47.8 mmHg vs. 46.5 mmHg) (Supplemental Fig. 1 C). The maximum selected logarithmic rank statistic was applied to binary the external validation cohort based on the predicted mean PG_AV_, and a clinical outcome similar to that of the internal derivation cohort was achieved (26.9% vs. 6.0%, *P* < 0.001).

## Discussion

The main findings of this study included the following: (i) The mean PG_AV_ levels assessed by TTE were consistently overestimated compared to the LHC measurements, and there was a significant inverse correlation between the mean PG_AV_ measured by TTE and EOAi. (ii) After analysis by the XGBoost algorithm (using 8 parameters as input variables), the mean PG_AV_ level measured by TTE was significantly improved. (iii) Cohesive hierarchical clustering algorithm analysis showed that cluster I patients had poorer cardiac function and a worse prognosis than those in cluster II. (iv) Patients with predicted mean PG_AV_ >68.6 mmHg had a significantly higher incidence of 2-year composite end points (*P* < 0.001). This finding may have potential prognostic value in patients with AS and a small annulus compared to the PPM definition under EOAi assessment (Central Illustration).

### Technical rationality of PG_AV_ correction

With the technological development and device iteration of TAVR, the indications have been extended to low-risk populations [16, 17]. However, a smaller annulus and body size are considered the strongest clinical predictors of PPM [5]. The ideal PG_AV_ after the procedures should be close to zero [18]. If the postprocedural PG_AV_ level is high, it may lead to poor hemodynamic improvement and increased left ventricular afterload, thus affecting the recovery of cardiac function and reducing the quality of the long-term prognosis [19, 20]. Previous studies by Ternacle et al. have shown that the PG_AV_ level is one of the important indicators for assessing EOAi and PPM classification [6]. However, for the small annuli, there are differences in the hemodynamic parameters measured by echocardiography and the intraprocedural invasive method, and the prognostic significance of the differences remains unclear [21]. There is a need to provide clinical corrective means developed in the field of TAVR to bridge the differences between the two and improve the accuracy of echocardiographic results. Therefore, quantitative identification of differences in PG_AV_ measurements between TTE and LHC is essential for preprocedural evaluation, procedural plan formulation, and prognosis improvement.

To the best of our knowledge, this study developed the first ML algorithm to improve TTE hemodynamic parameters for patients with AS with small annuli. The prediction model based on the XGBoost algorithm has several advantages. First, 8 parameters were aggregated based on the initial assessment of TTE and CTA as the strongest factors for improving the mean PG_AV_ level. Meanwhile, the mean PG_AV_ and EOAi values measured by TTE were the strongest predictors. Notably, we included parameters available to CTA in this study so that patients would not be exposed to invasive and potentially dangerous LHC in order to minimize the complex bias introduced by the algorithm. In the external validation cohort, the availability of the prediction model was also confirmed.

### Prognostic value and clinical application of corrected PG_AV_

Notably, we carried out the cohesive hierarchical clustering algorithm analysis for the internal derivation and external validation cohorts based on the clinical data of the composite end points. There were significant differences in physical state, especially in cardiac function, after the cluster differentiation. Leone et al. showed that more than moderate mitral regurgitation was an independent predictor of all-cause mortality in such patients. NT-proBNP levels may accelerate the functional decline of AV, thus significantly affecting the recovery of left ventricular function [1]. We also obtained similar results after cluster analysis. Previous studies have shown that PPM is associated with improved cardiac function, reduced resolution of left ventricular diastolic dysfunction, and increased risk of readmission for heart failure [22, 23]. Correspondingly, in our study, cardiac output and cardiac index were also significantly lower in cluster I patients (all *P* < 0.001).

Importantly, we used 68.6 mmHg as the predicted mean PG_AV_ cutoff using the maximum selected logarithmic rank statistic; also, the dichotomy of the cohort population to redefine PPM may have potential clinical practice implications. Previous studies have shown that PPM may reduce overall survival [13, 24] and seems to have an impact on mortality only when it exceeds a critical threshold [25, 26]. Paradoxically, some studies have also shown that the long-term mortality of PPM patients is similar to that of patients without PPM [27, 28]. To address this challenge, our ML algorithm-based mean PG_AV_ prediction model may help identify those patients who have the greatest chance of successfully extending survival after the procedures. Therefore, this model can be implemented in the periprocedural period to provide relevant information on cardiac function and prognosis after TAVR treatment on the basis of the accurate prediction of mean PG_AV_ levels to further improve clinical outcomes.

### Study limitations

This study has several limitations. First, although TTE and CTA parameters were analyzed in our study, we cannot rule out other unexamined variables that may further improve the availability of the predictive model. Therefore, it is essential to carry out large prospective studies in the future. Second, we excluded patients with extremely low PG_AV_ (mean PG_AV_ < 20 mmHg) in the study. Considering that cardiac pathophysiological changes in this population may cause unpredictable and complex effects on TTE measurements that will negatively affect the reliability and stability of the model, further studies are needed to improve the evaluation and prediction of such patients. In addition, our proposed prediction of a mean PG_AV_ level of ≥ 68.6 mmHg is intended only to provide a basis for assessing the greatest chance of a successful extension of survival in patients with a small annulus after the procedures; cardiac function and AV morphological changes under the PPM definition cannot be replaced.

## Conclusions

We analyzed the correlation and the differences of the mean PG_AV_ between TTE and LHC measurements and developed the first ML model to improve the accuracy of the mean PG_AV_ in patients with AS who have a small annulus. Importantly, patients with predicted elevated mean PG_AV_ levels showed increased mortality after the procedures, and the model was confirmed in the external validation cohort to be usable for prognostic risk estimates in patients with small annuli.

## Supplementary Information

Below is the link to the electronic supplementary material.

Supplementary file1

## Data Availability

The original contributions presented in the study are included in the article/supplementary material. Further inquiries can be directed to the corresponding author.

## Abbreviations

AS: Aortic stenosis
AV: Aortic valve
CTA: Computed tomography angiography
EOA: Effective orifice area
EOAi: Indexed EOA
LHC: Left heart catheterization
LVEF: Left ventricular ejection fraction
LVOT: Left ventricular outflow tract
ML: Machine learning
NT-proBNP: N-terminal pro-B-type natriuretic peptide
NYHA: New York Heart Association
PG_AV_: Pressure gradient of the aortic valve
PPM: Prosthesis–patient mismatch
TAVR: Transcatheter aortic valve replacement
THV: Transcatheter heart valves
TTE: Transthoracic echocardiography
XGBoost: Extreme gradient boost
