# Supplementary material for: Clinical significance of machine learning algorithm in predicting PPM during TAVR in small annuli
Source: Cardiovasc Interv Ther. 2026 Jan 5;41(2):402–13. doi: 10.1007/s12928-025-01215-5 (PMC13002776; doi:10.1007/s12928-025-01215-5)
Supplement: Supplementary file 1 — Supplementary file1 [file 12928_2025_1215_MOESM1_ESM.docx]

**Supplemental Materials**


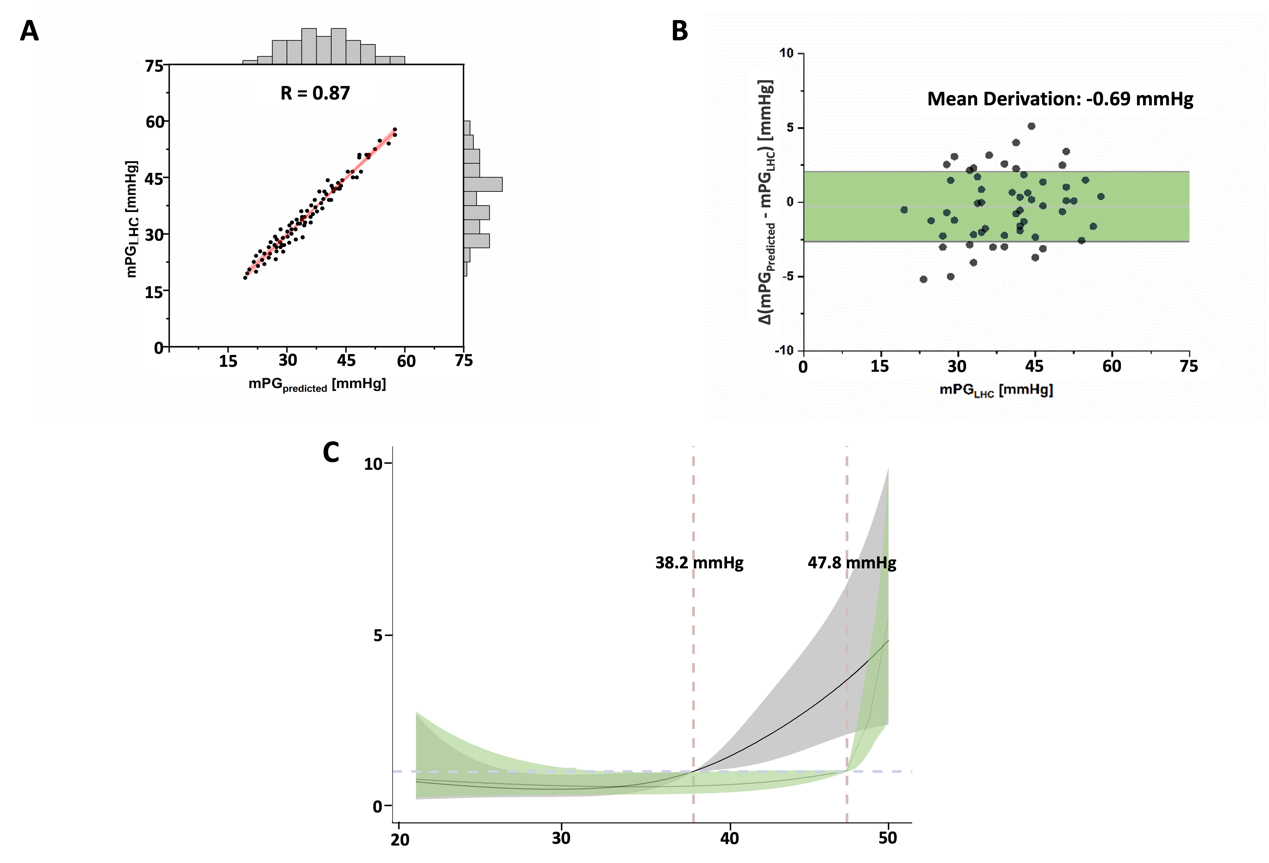


**SUPPLEMENTAL FIGURE 1.** **External validation.**

**(**A) There was a significant positive correlation between the predicted mean PG_AV_ and that obtained from the LHC measurements; (B) the mean PG_AV_ difference between them was -0.69 [95% confidence interval: -2.63, 2.38] mmHg. (C) The mean PG_AV_ cutoff values corresponding to moderate and severe PPM were 28.2 mmHg and 37.8 mmHg, respectively.

**SUPPLEMENTAL TABLE 1.** Baseline characteristics of the external validation cohort

|  | All Cohort (n = 118) | Cluster III (n = 42) | Cluster IV (n = 76) | *P*-Value |
| --- | --- | --- | --- | --- |
| Age, years | 75.0 (68.0–80.0) | 74.0 (67.0–79.5) | 76.0 (66.0–80.0) | 0.280 |
| Male | 51.7 (61) | 54.8 (23) | 50.0 (38) | 0.388 |
| Body mass index, kg/m^2^ | 25.0 (23.0–26.0) | 25.0 (22.0–29.0) | 25.00 (23.0–26.5) | 0.709 |
| Body surface area, m^2^ | 1.68 (1.59–1.78) | 1.68 (1.52–1.77) | 1.67 (1.56–1.77) | 0.497 |
| Diabetes mellitus | 22.8 (27) | 23.8 (10) | 22.4 (17) | 0.580 |
| Hypertension | 83.1 (98) | 83.3 (35) | 82.9 (63) | 0.887 |
| Dyslipidemia | 25.4 (30) | 26.2 (11) | 25.0 (19) | 0.508 |
| Peripheral artery disease | 10.2 (12) | 11.9 (5) | 9.2 (7) | 0.413 |
| COPD | 7.6 (9) | 7.1 (3) | 7.9 (6) | 0.462 |
| Cerebrovascular disease | 6.8 (8) | 7.1 (3) | 6.6 (5) | 0.835 |
| Chronic kidney disease | 11.9 (14) | 11.9 (5) | 11.8 (9) | 1.000 |
| Coronary artery disease | 29.7 (35) | 31.0 (13) | 28.9 (22) | 0.773 |
| Myocardial infarction | 5.1 (6) | 4.8 (2) | 5.3 (4) | 0.668 |
| Previous PCI | 23.7 (28) | 26.2 (11) | 22.4 (17) | 0.158 |
| Previous CABG | 6.8 (8) | 9.5 (4) | 5.3 (4) | 0.367 |
| Atrial ﬁbrillation | 13.6 (16) | 16.7 (7) | 11.8 (9) | 0.478 |
| Previous PPI | 5.9 (7) | 7.1 (3) | 5.3 (4) | 0.736 |
| NYHA functional class ≥ III | 87.3 (103) | 100.0 (42) | 80.3 (61) | **0.020** |
| STS score, % | 5.20 (3.40–7.40) | 5.50 (4.10–7.80) | 4.85 (3.60–7.10) | 0.601 |
| NT-proBNP, pg/mL | 1855.0 (1170.0–2442.0) | 2582.0 (1444.0 to 3371.0) | 1591.5 (1001.0 to 1771.5) | **< 0.001** |

CABG: coronary artery bypass grafting; COPD: chronic obstructive pulmonary disease; NT-proBNP, N-terminal pro-B-type natriuretic peptide; NYHA: New York Heart Association; STS: Society of Thoracic Surgeons; PCI: precancerous coronary intervention; PPI: permanent pacemaker implant.

**SUPPLEMENTAL TABLE 2.** Preprocedural imaging assessments of the external validation cohort

|  | All Cohorts (n = 118) | Cluster III (n = 42) | Cluster IV (n = 76) | *P*-Value |
| --- | --- | --- | --- | --- |
| **TTE and LHC** | | | | |
| Bicuspid aortic valve | 9.3 (11) | 11.9 (5) | 7.9 (6) | 0.712 |
| EOA, cm^2^ | 0.61 (0.49–0.75) | 0.60 (0.51–0.71) | 0.65 (0.51–0.74) | 0.727 |
| EOAi, cm^2^/m^2^ | 0.46 (0.35–0.56) | 0.45 (0.34–0.56) | 0.52 (0.37–0.61) | 0.800 |
| ≥ Severe aortic stenosis | 83.9 (99) | 88.1 (37) | 81.6 (62) | 0.116 |
| Combined with ≥ Moderate aortic regurgitation | 7.6 (9) | 11.9 (5) | 5.3 (4) | 0.381 |
| Combined with≥ Moderate mitral regurgitation | 11.9 (14) | 19.1 (8) | 7.9 (6) | **< 0.001** |
| Mean PG_AV_ measured by TTE, mmHg | 53.0 (47.5–58.0) | 54.5 (49.0–60.0) | 52.0 (47.0–58.0) | 0.385 |
| Mean PG_AV_ measured by LHC, mmHg | 42.0 (37.0–46.0) | 43.0 (37.5–45.0) | 41.5 (36.5–45.5) | 0.277 |
| LVEF, % | 51.0 (47.0–56.0) | 48.0 (44.0–54.0) | 54.0 (48.0–57.5) | 0.360 |
| Peak velocity, m/s | 3.7 (3.0–4.8) | 3.9 (3.5–4.4) | 3.4 (3.0–4.3) | 0.744 |
| LVEDV, mL | 77.0 (52.0–86.0) | 68.0 (62.0–98.5) | 83.0 (67.0–101.0) | 0.173 |
| LVESV, mL | 30.5 (19.0–39.0) | 24.0 (16.0–40.0) | 32.0 (18.0–51.0) | 0.679 |
| Left ventricular mass index, g/m^2^ | 128.0 (108.0–136.0) | 120.0 (112.5–149.5) | 135.0 (120.0–153.0) | 0.157 |
| Left atrial volume index, mL/m^2^ | 38.9 ± 7.1 | 38.8 ± 7.4 | 41.7 ± 6.8 | 0.125 |
| Cardiac output, L/min | 3.50 (2.83–4.59) | 3.43 (2.82–4.05) | 3.67 (3.35–4.04) | 0.800 |
| Cardiac index, L/min·m^2^ | 2.06 (1.66–2.38) | 2.03 (1.74 – 2.70) | 2.15 (1.89–2.62) | 0.538 |
| **Computed tomography angiography** | | | | |
| Annular area, mm^2^ | 345.0 ± 25.8 | 342.3 ± 28.4 | 351.3 ± 25.7 | 0.535 |
| Mean annular diameter, mm | 21.5 (20.6–22.5) | 21.0 (20.2–22.1) | 21.8 (20.7–22.4) | 0.320 |
| Minimum annular diameter, mm | 18.8 (17.6–19.9) | 18.6 (17.9–19.4) | 18.9 (17.8–20.4) | 0.661 |
| Maximum annular diameter, mm | 23.9 (22.5–25.0) | 23.1 (22.1–24.2) | 24.5 (22.9–24.9) | 0.125 |
| Annular perimeter, mm | 66.8 (64.4–69.5) | 65.8 (63.6–69.7) | 68.7 (66.2–69.8) | 0.435 |
| Annular ellipticity | 1.25 (1.14–1.34) | 1.26 (1.15–1.30) | 1.24 (1.17–1.36) | 0.897 |
| Area derived annular diameter, mm | 20.9 (19.8–21.5) | 20.6 (19.9–21.8) | 21.0 (20.2–21.9) | 0.751 |
| Perimeter derived annular diameter, mm | 21.0 (20.1–21.9) | 20.7 (20.2–21.8) | 21.2 (20.3–22.0) | 0.778 |
| Sinotubular junction diameter, mm | 26.1 (24.5–27.4) | 25.1 (23.9–27.2) | 26.2 (25.0–27.9) | 0.291 |
| LVOT diameter, mm | 29.2 (28.1–30.5) | 29.0 (27.4–30.2) | 29.8 (28.5–30.8) | 0.287 |
| Ascending aorta diameter, mm | 32.0 (29.8–34.4) | 31.1 (29.4–33.7) | 33.3 (30.1–35.2) | 0.413 |
| Left coronary artery height, mm | 11.9 (9.5–13.1) | 11.6 (10.2–13.1) | 12.0 (10.2–13.2) | 0.715 |
| Right coronary artery height, mm | 14.5 (12.7–15.5) | 13.9 (12.8–15.3) | 14.8 (12.9–15.9) | 0.570 |

EOA: effective orifice area; EOAi: indexed effective orifice area; LHC: left heart catheterization; LVEDV: left ventricular end diastolic volume; LVEF: left ventricular ejection fraction; LVESV: left ventricular end systolic volume; LVOT: left ventricular outflow tract; PG_AV_: pressure gradient of aortic valve; TTE: transthoracic echocardiography.

**SUPPLEMENTAL TABLE** **3.** Procedural details and in-hospital clinical outcomes of the external validation cohort

|  | All Cohorts (n = 118) | Cluster III (n = 42) | Cluster IV (n = 76) | *P*-Value |  |  |  |  |
| --- | --- | --- | --- | --- | --- | --- | --- | --- |
| **Procedural details** | | | | |  |  |  |  |
| Procedural success | 94.9 (112) | 92.9 (37) | 96.1 (73) | 0.777 |  |  |  |  |
| THV type | | | | |  |  |  |  |
| Self-expandable valve | 83.9 (99) | 81.0 (34) | 85.5 (65) | 0.372 |  |  |  |  |
| Balloon-expandable valve | 16.1 (19) | 19.1 (8) | 14.5 (11) | 0.653 |  |  |  |  |
| Predilation | 83.9 (99) | 71.4 (30) | 90.8 (69) | 0.124 |  |  |  |  |
| Postdilation | 17.8 (21) | 16.7 (7) | 18.4 (14) | 0.720 |  |  |  |  |
| Conversion to SAVR | 1.7 (2) | 4.8 (2) | 0 (0) | 1.000 |  |  |  |  |
| Malpositioning | 1.7 (2) | 2.4 (1) | 0 (0) | 1.000 |  |  |  |  |
| Annular rupture | 1.7 (2) | 2.4 (1) | 0 (0) | 1.000 |  |  |  |  |
| Device displacement | 5.9 (7) | 7.1 (3) | 5.3 (4) | 1.000 |  |  |  |  |
| Valve-in-valve implant | 5.9 (7) | 7.1 (3) | 5.3 (4) | 1.000 |  |  |  |  |
| Oversizing derived by perimeter ≥ 15% | 58.5 (69) | 57.1 (24) | 59.2 (45) | 0.591 |  |  |  |  |
| Immediate postprocedural mean PG_AV_ measured by LHC | -3.0 (-9.0–4.0) | 7.0 (3.0–12.0) | -17.0 (-24.0–9.5) | **< 0.001** |  |  |  |  |
| **In-hospital clinical outcomes** | | | | |  |  |  |  |
| All-cause deaths | 1.7 (2) | 4.8 (2) | 0 (0) | 1.000 |  |  |  |  |
| Major adverse cardiovascular events | 1.7 (2) | 4.8 (2) | 0 (0) | 1.000 |  |  |  |  |
| Stroke | 0.9 (1) | 2.4 (1) | 0 (0) | 1.000 |  |  |  |  |
| Life-threatening bleeding | 3.4 (4) | 4.8 (2) | 2.6 (2) | 1.000 |  |  |  |  |
| Acute kidney failure | 5.1 (6) | 7.1 (3) | 4.0 (3) | 1.000 |  |  |  |  |
| Major vascular complications | 3.4 (4) | 7.1 (3) | 1.3 (1) | 1.000 |  |  |  |  |
| New-onset PPI | 7.6 (9) | 16.7 (6) | 4.0 (3) | **< 0.001** |  |  |  |  |
| Postprocedural mean PG_AV_ measured by TTE | 10.5 (8.0–16.0) | 18.0 (8.0–25.0) | 6.0 (2.0–9.0) | **< 0.001** |  |  |  |  |
| Postprocedural EOAi measured by TTE | 0.88 (0.68–0.97) | 0.72 (0.59–0.88) | 1.06 (0.93–1.20) | **< 0.001** |  |  |  |  |

EOAi: indexed effective orifice area; LHC: left heart catheterization; PGAV: pressure gradient measured by transthoracic echocardiography; PPI: permanent pacemaker implant; SAVR: surgical aortic valve replacement; THV: transcatheter heart valve; TTE: transthoracic echocardiography.
